# Laparoscopic assisted versus ultrasound guided transversus abdominis plane block in laparoscopic bariatric surgery: a randomized controlled trial

**DOI:** 10.1186/s12871-024-02498-6

**Published:** 2024-04-06

**Authors:** Mohammad Fouad Algyar, Karim Sabry Abdelsamee

**Affiliations:** 1 Anesthesiology, Surgical Intensive Care and Pain Medicine Department, Faculty of Medicine, Kafr ElSheikh University, Kafr ElSheikh, 33516 Egypt; 2 Surgery Department, Faculty of Medicine, Ain Shams University, Cairo, Egypt

**Keywords:** Nerve block, Laparoscopy, Ultrasonography, Bariatric surgery, Obesity

## Abstract

**Background:**

Transversus abdominis plane block (TAPB) guided by laparoscopy and ultrasound showed promise in enhancing the multimodal analgesic approach following several abdominal procedures. This study aimed to compare the efficacy and safety between Laparoscopic (LAP) TAP block (LTAP) and ultrasound-guided TAP block (UTAP) block in patients undergoing LAP bariatric surgery.

**Patients and methods:**

This non-inferiority randomized controlled single-blind study was conducted on 120 patients with obesity scheduled for LAP bariatric surgeries. Patients were allocated into two equal groups: LTAP and UTAP, administered with 20 mL of 0.25% bupivacaine on each side.

**Results:**

There was no statistically significant difference in the total morphine consumption, Visual Analogue Scale (VAS) score at all times of measurements, and time to the first rescue analgesia (*p* > .05) between both groups. The duration of anesthesia and duration of block performance were significantly shorter in the LTAP group than in the UTAP group (*p* < .001). Both groups had comparable post-operative heart rate, mean arterial pressure, adverse effects, and patient satisfaction.

**Conclusions:**

In LAP bariatric surgery, the analgesic effect of LTAP is non-inferior to UTAP, as evidenced by comparable time to first rescue analgesia and total morphine consumption with similar safety blocking through the low incidence of post-operative complications and patient satisfaction.

**Trial registration:**

The study was registered in Pan African Clinical Trials Registry (PACTR) (ID: PACTR202206871825386) on June 29, 2022.

## Background

Bariatric surgery represents the most promising management in patients with obesity for weight reduction and improvement of the associated comorbidities compared to other methods, such as non-surgical intervention, behavior modification, and diet therapy [1, 2].

Although laparoscopic (LAP) bariatric surgery is minimally invasive, post-operative pain ranges from mild to severe. A greater risk of respiratory depression generated by opioids may make it challenging to manage pain in morbidly patients with obesity [3]. Pain control can be achieved with methods such as Intravenous (IV) analgesics, local anesthetic infiltration to the wound site, and regional blocks such as the Transversus Abdominis Plane (TAP) block [4, 5].

TAP has developed as a simple, safe, and affordable method adopted into several Enhanced Recovery After Surgery (ERAS) protocols to produce narcotic-sparing analgesia after bariatric surgery, improve post-operative measurements, and decrease the length of stay postoperatively and cost [6, 7].

Previous studies suggested using Ultrasound (US) for needle placement due to the risk of damaging the adjacent structures and decreasing the intravascular injection [8, 9].

The US approach may not be a reliable final endpoint as the Transversus Abdominis Muscle (TAM) is relatively thin. The local anesthetic may be administered above, in, or below the real TAP [10]. Magee et al. [11] administered a TAP block guided by LAP view before the surgical intervention to prevent iatrogenic complications.

Moreover, many surgeries have used Laparoscopic TAP (LTAP) block for analgesia, and significant results have been obtained [12, 13]. Ruiz-Tovar et al. [14] noted that LTAP block provides excellent analgesia following LAP gastric bypass.

US TAP and LTAP blocks were designed to help find the right plane and limit peritoneal invasion, but comparative studies between LTAP and UTAP techniques are still scarce [15–17].

We hypothesize that LTAP block is non-inferior to UTAP block in patients who underwent LAP bariatric surgery. This study aimed to compare the efficacy and safety of LTAP versus UTAP block following LAP bariatric surgery.

## Patients and methods

### Study design

Single-blinded, non-inferiority randomized controlled trial.

#### Study setting

Tertiary health care facility.

##### Recruitment center

General Surgery Department, Faculty of Medicine, Ain Shams University, From July 2022 to October 2022, after obtaining approval of the local ethics committee of Surgery Department's Institutional Ethics Committee, Ain Shams University, Cairo, Egypt, and registration on the Pan African Clinical Trials Registry (ID: PACTR202206871825386) on 29/06/2022.

#### Sampling method

Consecutive sampling technique was adopted [18].

## Sample Size

The minimal sample size is calculated based on a previous study aimed to compare LAP -assisted transversus abdominis plane (TAP) block with periportal local anaesthetic infiltration in managing post-operative pain. Mughal et al. [12] reported that their analysis had demonstrated the therapeutic benefit of LAP -assisted TAP block in initial post-operative pain management for patients undergoing elective LAP inguinal hernia repair. Based on their results, adopting a power of 80% to detect a standardized effect size (non-inferiority margin, d) of 1 in mean pain score (primary outcome), and level of significance 95% (α = 0.05), the minimum required sample size was found to be 60 patients per group (number of groups = 2) (Total sample size = 120 patients) [19, 20]. Any withdrawal for any reason was compensated by replacement to control for attrition (withdrawal) bias [21]. The sample size was calculated using online Power calculators Single blinded approach was adopted. Blinding was employed to participants [22].

The allocation sequence was generated using permuted block randomization technique, and the block size was variable [23]. Allocation sequence/code was concealed from the person allocating the participants to the intervention arms using sealed opaque envelopes [24]. Patients were allocated into two equal groups in a parallel manner: LTAP and UTAP groups. Patients were blinded by group allocation.

Written informed consent was obtained from the patients before participation. One hundred and twenty patients with obesity of either sex with ages ranging from 18 to 56 years, American Society of Anesthesiologists (ASA) class II or III, and body mass index (BMI) ≥ 35 kg/m^2^ who were scheduled LAP bariatric surgeries (Sleeve gastrectomy surgery).

Exclusion criteria were: prior laparotomy, BMI ≥ 60 kg/m^2^, history of cardiac arrhythmia, diagnosis of chronic pain syndrome, allergy to bupivacaine and current daily opioid usage, and history of alcohol or drug abuse during the last six months.

### Outcome measures

The primary outcomes were total post-operative opioid consumption and post-operative pain scores (using Visual Analogue Scale (VAS)) at rest and in movement. In contrast, the secondary outcomes were the time of the first analgesic requested by the patient and the surgical duration.

### Preoperative assessment and preparation

Clinical evaluations and standard laboratory tests were performed on all individuals. All participants were instructed about the VAS score for pain assessment.

Peripheral IV access was established under strict sterile circumstances upon arrival at the operative room. All patients were given pre-medication consisting of midazolam (2 to 3 mg IV) and metoclopramide (10 mg IV).

Perioperative monitoring included electrocardiogram (ECG), noninvasive blood pressure (NIBP), pulse oximetry, Capnogram, and temperature probe.

### Anesthetic techniques

The induction of general anesthesia was achieved using 1–2 µg/kg fentanyl (IV), 100mg lidocaine, and 2mg/kg propofol of ideal body weight + 40% weight excess. Air and oxygen with a FiO2 of 0.8 were used to maintain anesthesia while inhaling isoflurane at a concentration of 1.5–2%. A train of four of 0 was maintained using IV rocuronium to relax the patient's muscles during the surgery.

All surgical procedures were done by the same surgery team, who was qualified and expert in these operations.

Patients allocation:

Patients were randomly allocated into two equal groups:

- LTAP Group (*n* = 60)

Under direct vision, the operative surgeon inserted an 18G LAP needle between the lower costal border and the iliac crest at the midaxillary line via a working port until the "pop" was felt. Then, 2 mL of normal saline was administered to confirm the right position. 20 mL of 0.25% bupivacaine was injected into each side after noting Doyle's internal bulge sign, which is the bulge that forms when the TAM with the peritoneum is pushed inside.

- ULAP Group (*n* = 60)

The anesthesiologist conducted the UTAP blocks. A high-frequency linear transducer was positioned in the midaxillary line to measure the area between the iliac crest and the lower costal margin. After the TAM was identified, the anesthesiologist used in-plane US- guidance to insert a 22G Tuohy needle between the internal oblique and TAM, injecting 2 mL of saline to ensure proper needle placement before injecting 20 mL of 0.25% bupivacaine into each side.

### Post-operative assessment

After reversing muscular relaxation with neostigmine, Extubation was carried out in a semi-sitting position. The patient was transferred to the Post-Anethesia Care Unit (PACU) and administered paracetamol 1gm/6h IV as routine analgesia.

Heart rate (HR), mean arterial pressure (MAP), and VAS score were measured at PACU, 1h, 2h. 4h, 6h, 8h, 12h, 18h, and 24h post-operative. When the VAS score exceeded three, an analgesic dose of morphine 3 mg IV was administered. Time to the 1st rescue analgesic request, the total amount of rescue analgesic in 1st 24h post-operative, the incidence of adverse events [hypotension (MAP < 20% of baseline value) and bradycardia (HR < 60 beats/min), post-operative nausea and vomiting (PONV)] and patients' satisfaction score were recorded.

The primary outcome were post-operative pain scores and total post-operative opioid consumption. The secondary outcomes were the time of 1st analgesic requested by the patient and satisfaction.

### Statistical methodology

#### Statistical methodology

Data were collected and analyzed using Statistical Package for Social Science (SPSS) program (ver 25) [25].Kolmogorov–Smirnov test of normality was carried out [26]. Data were described using minimum, maximum, mean, standard deviation, standard error of the mean, and 95% CI of the mean for the normally distributed data and median, 95% CI of the median for the not-normally distributed data [27]. Independent unpaired sample t-test [28] with Welch's t-test correction [29] were used to compare quantitative parametric variables. Mann–Whitney U test [30] was used to compare quantitative non-parametric variables. Friedman's test [31], Post-hoc pair-wise comparison using Dunn-Sidak method [32] to compare repeated measures of quantitative non-parametric variables. One-way repeated measures analysis of variance [33] to compare repeated measures of quantitative parametric variables. Z-test for comparing different independent proportions was used. During sample size calculation, beta error accepted up to 20% with a power of study of 80%. An alpha level was set to 5% with a significance level of 95%. Statistical significance was tested at *p*-value < 0.05 [34].

## Results

### Participant flow

**Fig. 1:**
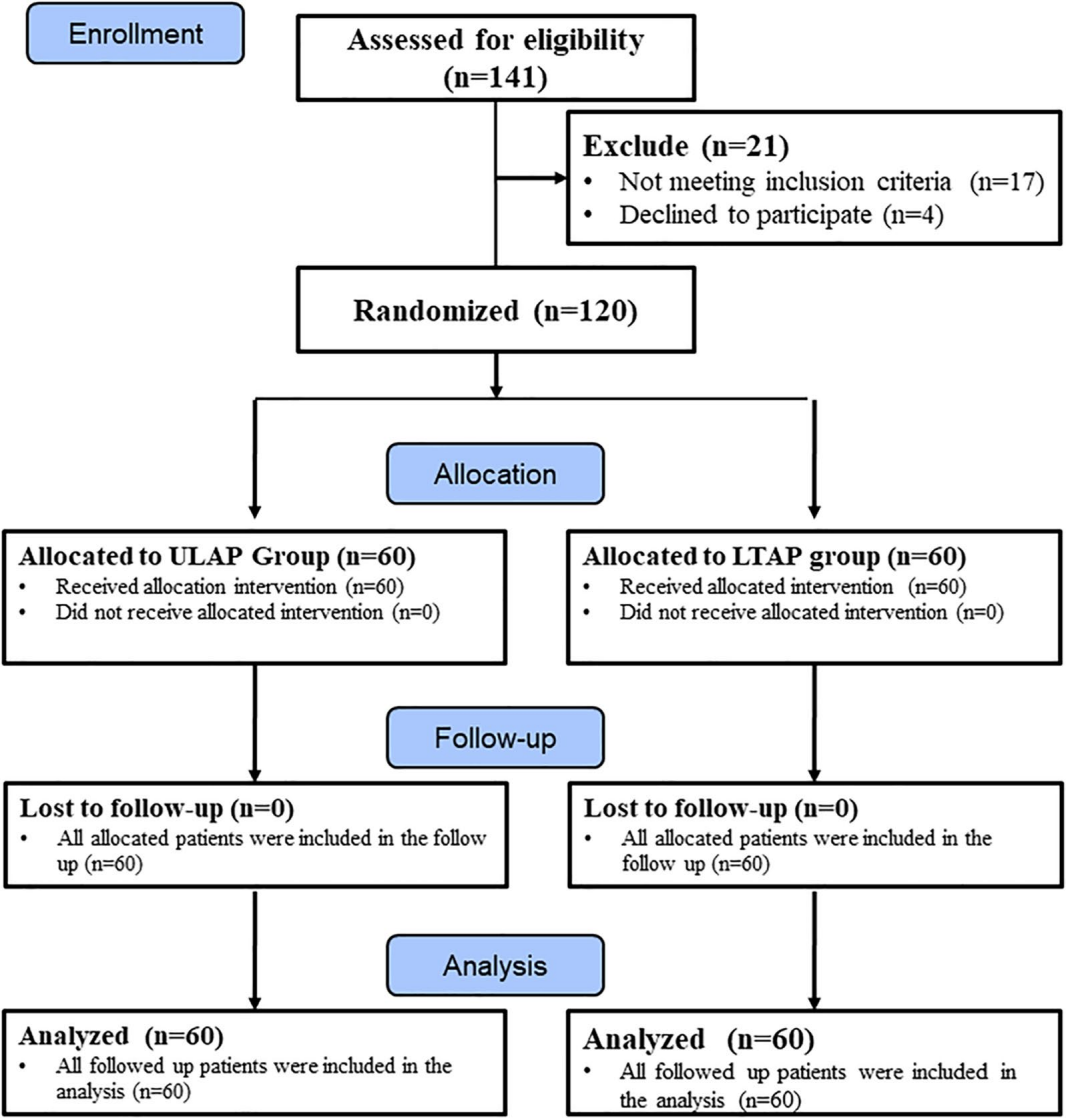
Consort flow diagram

One hundred forty-one patients were assessed for eligibility, 17 were excluded, and four declined to participate. The remaining 120 were recruited and randomly allocated (60 patients in the ULAP group and 60 in the LTAP group) and analyzed with no patient lost to follow-up (Fig. 1).

### Patients’ characteristics

There were no statistically significant differences in age (*p* = 0.114), sex (*p* = 0.459), weight (*p* = 0.259), height (*p* = 0.229), BMI (*p* = 0.519), WHO classification of BMI (*p* = 0.056), ASA classification (*p* = 0.315), duration of surgery (*p* = 0.516). The mean duration of anesthesia in the LTAP group (47.62 ± 4.27 min) was statistically significantly shorter than in the UTAP group (54.23 ± 5.60 min) (*p* < 0.001). The mean block performance in the LTAP group (2.8 ± 0.8min) was statistically significantly shorter than in the UTAP group (7.13 ± 1.76 min) (*p* < 0.001) (Table 1).

**Table 1:** Demographic data, duration of surgery, anesthesia and block performance in the two groups

	**Total (*n* = 120)**	**Group**		***p*-vaue**
		**UTAP (*n* = 60)**	**LTAP (*n* = 60)**	
**Age (years)**				
**Min–Max**	18.00–56.00	21.00–56.00	18.00–56.00	t_(W)(df=106.835)_ = 1.595
**Mean ± SD**	31.08 ± 8.61	32.23 ± 7.20	29.92 ± 9.74	*p* = .114 NS
**SE of Mean**	0.79	0.93	1.26	
**95.0% CI of the mean**	29.52–32.63	30.37–34.09	27.40–32.43	
**Sex**				
**Male**	50 (41.67%)	23 (38.33%)	27 (45.00%)	χ^2^_(df=1)_ = 0.549
**Female**	70 (58.33%)	37 (61.67%)	33 (55.00%)	*p* = .459 NS
**Sex ratio (male: female ratio)**	71.4%	62.1%	81.8%	*p* = .235
**Weight (kg)**				
**Min–Max**	82.00–137.00	88.00–120.00	82.00–137.00	t_(W)(df=99.859)_ = 1.136
**Mean ± SD**	105.53 ± 10.76	104.42 ± 8.14	106.65 ± 12.83	*p* = .259 NS
**SE of Mean**	0.98	1.05	1.66	
**95.0% CI of the mean**	103.59–107.47	102.31–106.52	103.33–109.96	
**Height (meter)**				
**Min–Max**	1.46–1.82	1.53–1.70	1.46–1.82	t_(W)(df=99.953)_ = 1.209
**Mean ± SD**	1.62 ± 0.06	1.62 ± 0.05	1.63 ± 0.08	*p* = .229 NS
**SE of Mean**	0.01	0.01	0.01	
**95.0% CI of the mean**	1.61–1.64	1.60–1.63	1.61–1.65	
**BMI (kg/m**^**2**^**)**				
**Min–Max**	35.11–49.45	35.11–47.84	35.11–49.45	t_(df=118)_ = 0.647
**Mean ± SD**	40.00 ± 3.16	39.97 ± 3.06	40.03 ± 3.28	*p* = .519 NS
**SE of Mean**	0.29	0.39	0.42	
**95.0% CI of the mean**	39.43–40.57	38.18–40.76	39.18–40.88	
**WHO classification of BMI**				
**‘30.0–34.9: Obesity class I’**	18 (15.00%)	5 (8.33%)	13 (21.67%)	χ^2^_(df=2)_ = 5.832
**‘35.0–39.9: Obesity class II’**	50 (41.67%)	30 (50.00%)	20 (33.33%)	*p* = .056 NS
**‘40 or Above: Obesity class III’**	52 (43.33%)	25 (41.6%)	27 (45.00%)	
**ASA**				
**Two**	85 (70.83%)	40 (66.67%)	45 (75.00%)	χ^2^_(df=1)_ = 1.008
**Three**	35 (29.17%)	20 (233.33%)	15 (25.00%)	*p* = .315 NS
**Duration of surgery (min)**				
**Min–Max**	34.00–62.00	34.00–58.00	36.00–62.00	t_(W)(df=115.704)_ = 0.651
**Mean ± SD**	45.95 ± 5.72	46.3 ± 6.13	45.62 ± 5.32	*p* = .516
**SE of Mean**	0.52	0.79	0.68	
**95.0% CI of the mean**	-1.39–2.76	44.71–47.88	44.24–46.99	
**Duration of anesthesia (min)**				
**Min–Max**	40.00–65.00	44.00–65.00	40.00–61.00	t_(W)(df=110.277)_ = 7.277
**Mean ± SD**	50.93 ± 5.97	54.23 ± 5.60	47.62 ± 4.27	*p* < .001*
**SE of Mean**	0.54	0.72	0.55	
**95.0% CI of the mean**	49.85–52.00	52.79–55.68	46.51–48.72	
**Duration of block performance (min)**				
**Min–Max**	2.00–10.00	5.00–10.00	2.00–4.00	t_(W)(df=82.271)_ = 17.36
**Mean ± SD**	4.96 ± 2.56	7.13 ± 1.76	2.8 ± 0.8	*p* < .001*
**SE of Mean**	0.23	0.22	0.10	
**95.0% CI of the mean**	3.83–4.82	6.67–7.58	2.59–3.00	

*n* number of patients, *Min–Max* Minimum to Maximum, *SD* Standard deviation, *SE* Standard error, *CI* Confidence interval, *t* Independent Sample t test, *W* Welch's correction, χ^2^ Pearson Chi-Square, *df* degree of freedom, *NS* Statistically not significant (*p ≥ *05). *: Statistically significant (*p* < .05)

### Hemodynamic and respiratory variables

**Fig. 2:**
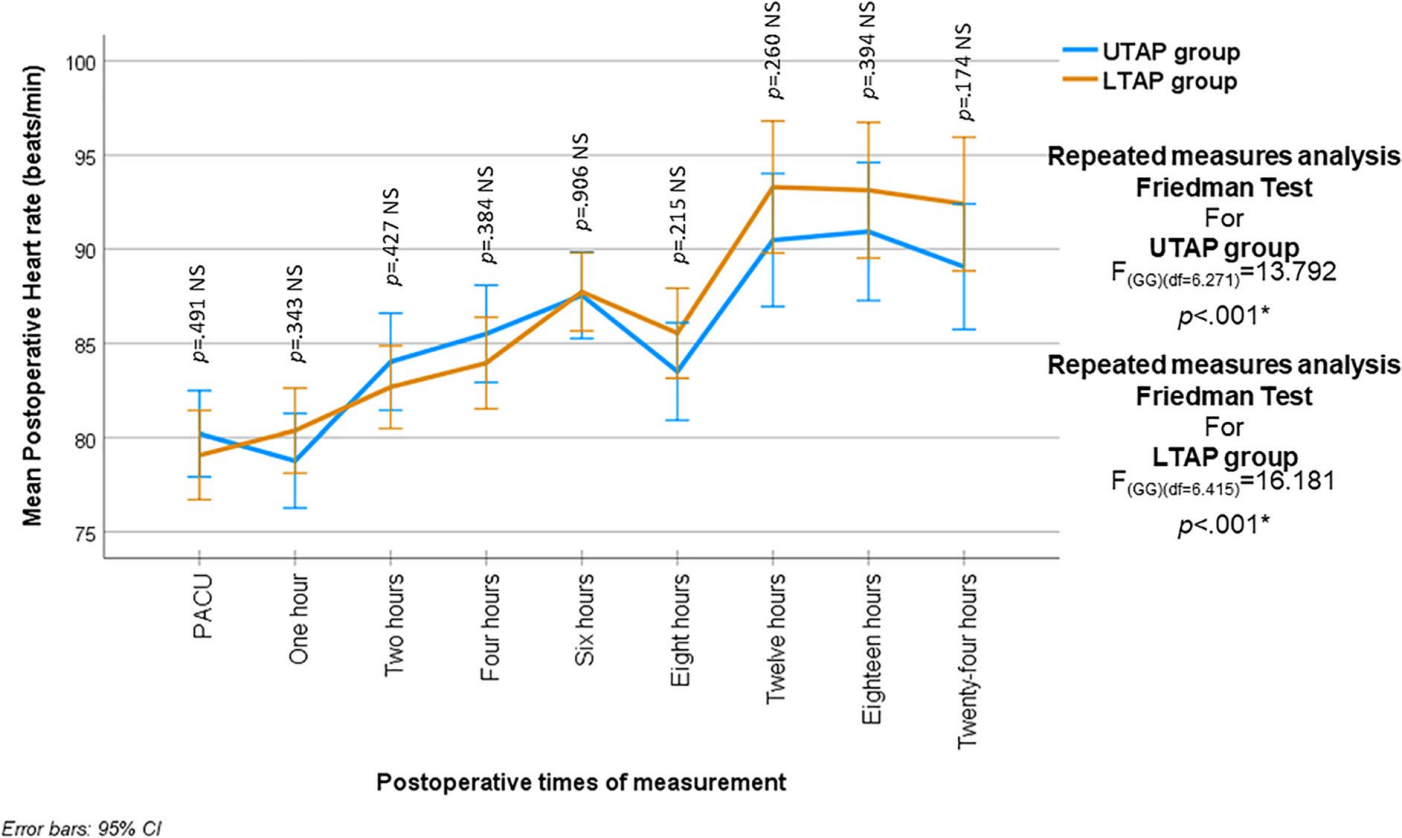
Mean (95% CI) postoperative heart rate (beats/min) in the two studied groups at all times of measurements

There was no statistically significant difference in the post-operative heart rate between the two groups during all times of measurement (*p* > 0.05). One-way repeated measures analysis in each group revealed a statistically significant change in heart rate among the different times of measurement (*p* < 0.001) (Fig. 2).

**Fig. 3:**
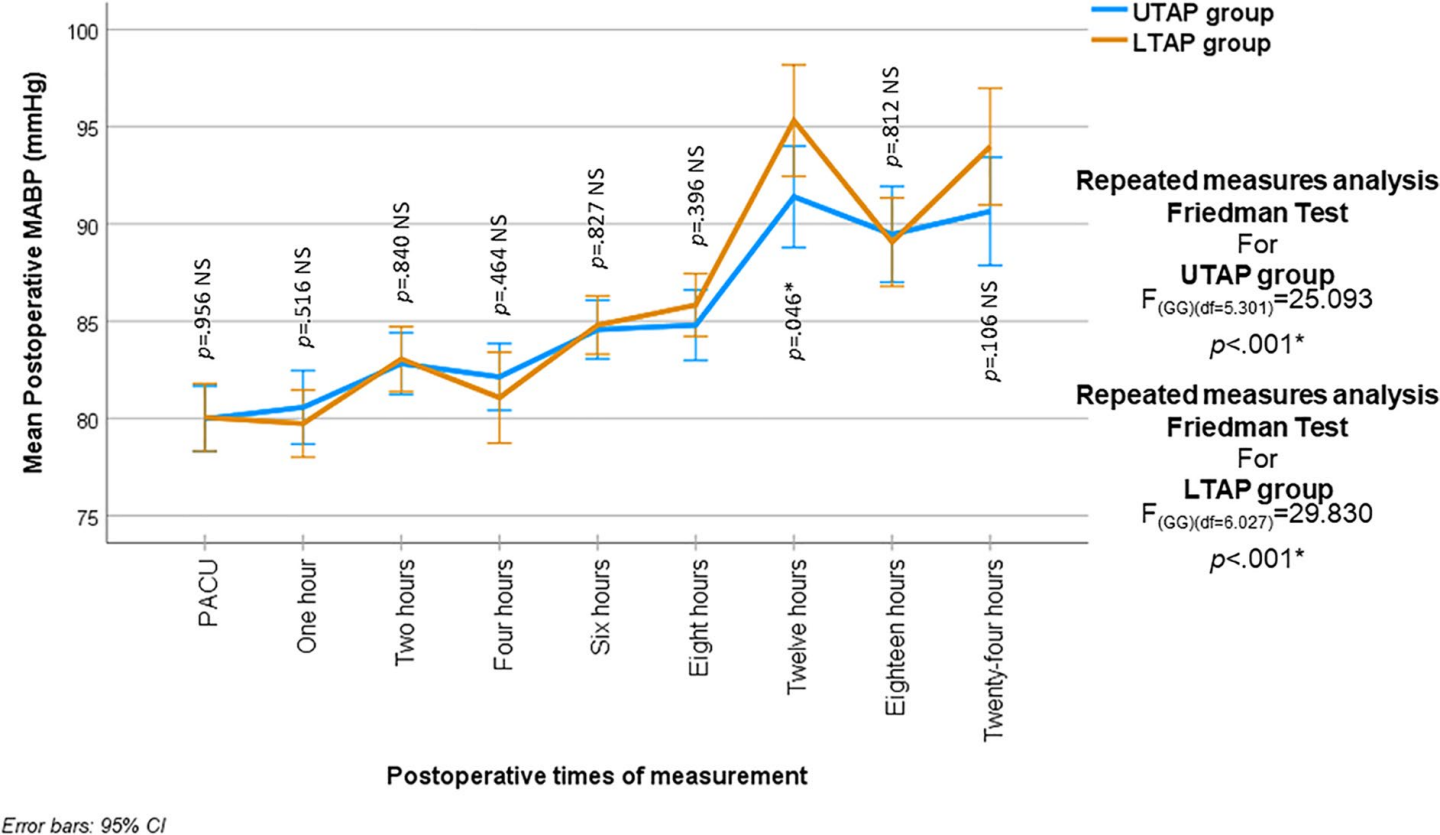
Mean (95% CI) postoperative mean arterial blood pressure (MABP) (mmHg) in the two studied groups at all times of measurements

There was no statistically significant difference in the post-operative Mean Arterial Blood Pressure (MABP) between the two groups during all times of measurement (*p* > 0.05) except at 12 h post-operative, where the MABP was statistically significantly higher in the LTAP group (95.32 ± 11.11 mmHg) when compared with UTAP group (91.40 ± 10.09 mmHg) (*p* = 0.046). One-way repeated measures analysis in each group revealed a statistically significant change in MABP among the different times of measurements (*p* < 0.001) (Fig. 3).

### Time to the first rescue analgesia

There was no statistically significant difference in time to the first rescue analgesia between the UTAP group (12.60 ± 6.43 h) when compared with the LTAP group (14.29 ± 5.84 h) (*p* = 0.384).

### Total morphine consumption

The mean of total morphine consumption is statistically not different between the UTAP group (1.22 ± 1.93 mg) and LTAP group (1.30 ± 1.94 mg) when calculated for all included patients (*n* = 60 per group) (*p* = 0.814). In the UTAP group, 20/60 (33.33%) needed morphine compared with 21/60 (35.00%) in the LTAP group, with no statistically significant difference (*p* = 0.849). Also, there was no statistically significant difference in the total morphine consumption between the two groups when calculated for only patients who need morphine (*p* = 0.848) (Table 2).

**Table 2:** Time to the first rescue analgesia, total morphine consumption, Postoperative complications, and patient satisfaction score in the two studied groups

	**Total**	**Group**		**Test of significance** ***p*-value**
		**UTAP**	**LTAP**	
**Time to the First Rescue Analgesia (hours)**				
*n*	41	20	21	t_(df=39)_ = 0.880
Min–Max	6.00–24.00	6.00–24.00	6.00–24.00	*p* = .384 NS
Mean ± SD	13.46 ± 6.12	12.60 ± 6.43	14.29 ± 5.84	
SE of Mean	0.96	1.44	1.27	
95.0% CI of the mean	11.53–15.39	9.59–15.61	11.63–16.94	
**Total Morphine consumption (mg)**				
*n*	120	60	60	t_(df=118)_ = 0.236
Min–Max	0.00–6.00	0.00–6.00	0.00–6.00	*p* = .814 NS
Mean ± SD	1.26 ± 1.93	1.22 ± 1.93	1.30 ± 1.94	
SE of Mean	0.18	0.25	0.25	
95.0% CI of the mean	0.91–1.61	0.72–1.72	0.80–1.80	
**Total Morphine consumption (for only patients who needed Morphine) (mg)**				
*n*	41	20 (33.33%)	21 (35.00%)	Z = 0.192, *p* = .849 NS
Min–Max	1.00–6.00	1.00–6.00	3.00–6.00	
Mean ± SD	3.68 ± 1.39	3.65 ± 1.50	3.71 ± 1.31	t_(df=39)_ = 0.147, *p* = .884 NS
SE of Mean	0.22	0.33	0.29	
95.0% CI of the mean	3.25–4.12	2.95–4.35	3.12–4.31	
**Postoperative findings:**				
Nausea and vomiting	18 (15.00%)	11 (18.33%)	7 (11.67%)	Z = 1.022, *p* = .307 NS
Hypotension	10 (8.33%)	6 (10.00%)	4 (6.67%)	Z = 0.660, *p* = .509 NS
Bradycardia	10 (8.33%)	6 (10.00%)	4 (6.67%)	Z = 0.660, *p* = .509 NS
**Patient satisfaction score**				
*n*	120	60	60	
Min–Max	3.00–5.00	3.00–5.00	3.00–5.00	
Mean ± SD	4.47 ± 0.69	4.25 ± 0.77	4.70 ± 0.50	t_(W)(df=100.717)_ = 3.792
SE of Mean	0.06	0.10	0.06	*p* < .001*
95.0% CI of the mean	4.35–4.60	4.05–4.45	4.57–4.83	

*n* number of patients, *Min–Max* Minimum to Maximum, *SD* Standard deviation, *SE* Standard error, *CI* Confidence interval, *t* Independent Sample t test, *W* Welch's correction, χ^2^ Pearson Chi-Square, *df* degree of freedom, *NS* Statistically not significant (*p*** ≥ **.05). *: Statistically significant (p < .05)

### Visual analogue scale (VAS) score

**Fig. 4:**
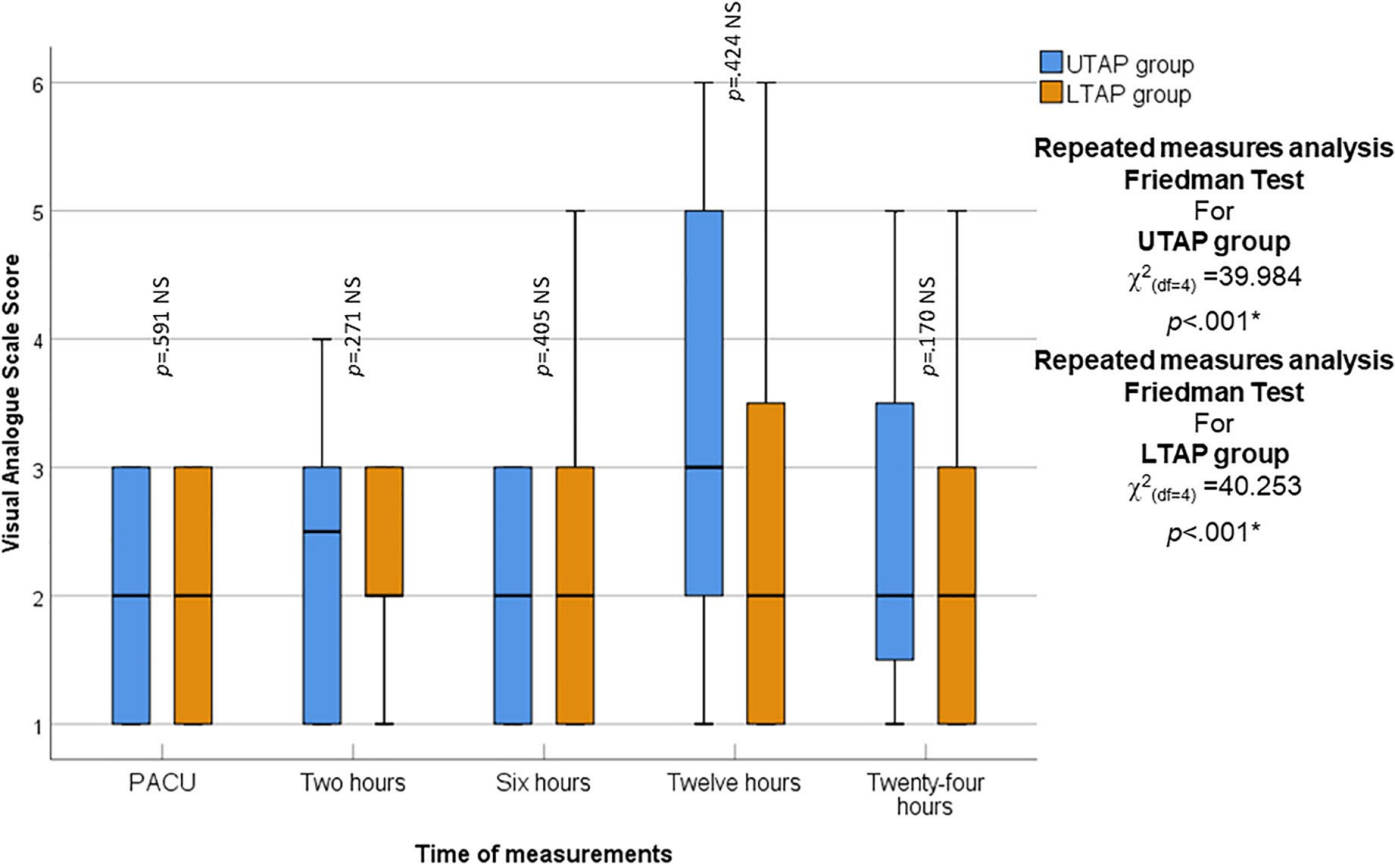
Box and whisker graph of Visual Analogue Scale score in the studied groups, the thick line in the middle of the box represents the median, the box represents the inter-quartile range (from 25 to 75th percentiles), the whiskers represent the minimum and maximum at all times of measurements

There was no statistically significant difference in Post-operative Pain by Visual Analogue Scale (VAS) score for pain between the two groups during all times of measurement (*p* > 0.05). One-way repeated measures analysis revealed a statistically significant change in VAS score among the different times of measurements in both the UTAP group and LTAP (*p* < 0.001, *p* = 0.001; respectively) (Fig. 4).

### Incidence of post-operative adverse effect

The incidence of adverse effects was not significantly different between both groups. No patients developed any signs of local anesthetic toxicity. There was no statistically significant difference between both groups as regards post-operative nausea and vomiting (*p* = 0.307), post-operative hypotension (*p* = 0.509), and post-operative bradycardia (*p* = 0.509) (Table 2).

### Patient satisfaction score

In the LTAP group, the patient satisfaction score (4.70 ± 0.50) was statistically significantly higher when compared with the UTAP group (4.25 ± 0.77) (*p* < 0.001) (Table 2).

## Discussion

Bariatric surgeries are considered the most effective treatment option for weight reduction. However, achieving adequate pain control in these patients is challenging because of an increased predisposition to opioid-induced respiratory depression [35]. Post-operative pain inhibits respiration, and ambulation, resulting in cardiovascular, thromboembolic, and pulmonary complications associated with an increased mortality risk [36].

Three different processes affect the development of pain after LAP surgery. 50–70% of the overall pain is categorized as parietal discomfort due to abdominal wall damage caused by port insertion. Also, 20–30% of the total discomfort comes from irritated diaphragms caused by pneumoperitoneum formation, and 10–20% of discomfort is attributed to visceral pain caused by LAP manipulation of the stomach and small intestine [35].

The drawbacks of laparoscopy TAP include: When inserted in a blind fashion, it carries a risk of visceral injury [37]. To avoid complications, ultrasound-guided transversus abdominis plane block technique (UTAP) was adopted by anesthesiologists.

The TAP block was commonly used for post-operative analgesia that lowers opioid consumption for patients with obesity [35]. The TAP technique has evolved and can be guided by either ultrasound or LAP guidance [38].

The main findings in our study indicated that LTAP was non-inferior to UTAP in providing post-operative analgesia (comparable VAS score, time to 1st rescue analgesia, and morphine requirement), and patient satisfaction score in patients with obesity undergoing bariatric surgeries but with significantly shorter surgical time in LTAP group than the UTAP group. In addition, hemodynamic measurements and associated adverse effects were comparable in both groups.

TAP block is a locoregional anaesthetic approach that blocks the anterolateral abdominal wall's nerve supply, and it was shown to decrease post-operative pain and morphine needed after major abdominal surgeries [39]. This fascial plane field block between the internal oblique and TAM affects the lower thoracic and lumbar spinal neural afferents [40].

UTAP blocks are efficient and result in satisfactory immediate post-operative analgesia in bariatric surgeries, as reported by Sun et al. [41] and Sinha et al. [42], who noted that the UTAP block group showed perioperative opioid savings with an improvement in the UTAP group's VAS score compared to the control group. Also, Emile et al. [43], Mittal et al. [44], and Wasef and colleagues [45] reported lower pain scores in patients who received TAP block with lower opioid consumption and PONV than controls. Patients in the TAP block group reported higher satisfaction with the operation overall and pain management after discharge.

Additionally, Sinha et al. [42] indicated the efficacy of the UTAP multimodal analgesic method in patients having LAP gastric bypass, as measured by decreased opioid demand, improved pain score, reduced sedation, early ambulation, and increased patient satisfaction.

UTAP block is one of the most common techniques used in bariatric surgeries. Chetwood et al. [10] identified LTAP provided less time-consuming and may be done exclusively by surgeons without needing US experience in addition to enhanced pain relief, early discharge, lower hospital stay, and total care costs. However, the US may not be a potential option in all medical centers; hence, LAP guidance may serve as an alternative where surgeons can apply the block without depending on the US using a LAP camera [46].

Moreover, Seiler et al. [47] reported a significant reduction in IV opioid use postoperatively in the LTAP group compared with the control group in all post-operative measurements for morbidly patients with obesity after LAP Roux-en-Y gastric bypass surgery.

Tihan et al. [48] and Said et al. [49] indicated a significant reduction in post-operative pain and PONV scores in LTAP block up to 24 h post-operative. The analgesic effect of LTAP was also demonstrated by Favuzza and Delaney [46], who declared that the LATP block had efficient pain relief for abdominal incisions, reduced narcotic use, and short hospital stay in patients who had LAP colorectal surgery.

Previous studies compared LTAP and UTAP block in patients getting minimally invasive surgeries; nevertheless, outcomes were contradictory.

Our results align with Park et al. [50], who demonstrated that LTAP was non-inferior to UTAP as there was no significant difference in pain score and post-operative morphine consumption between the two groups following colorectal surgeries.

Moreover, Wong et al. [51] reported no significant difference in post-operative narcotic consumption with similar pain scores between LTAP and UTAP blocks in all time post-operative measurements in patients who underwent LAP colorectal surgery.

Similarly, Sahap et al. [52] reported no significant difference between the LTAP and UTAP groups regarding hemodynamic measurements, tramadol consumption, VAS score, and PONV in the post-operative period in patients who underwent LAP cholecystectomy.

In contrast to our results, Zaghiyan et al. [53] observed that at 24 h postoperatively, LTAP block was better than UTAP block in pain management and opioid needs. The discrepant results may be due to the different surgical procedures, patient's characteristics, and different volumes of local anaesthetic used in addition to the usage of epinephrine.

Although our study has some strengths, there are a few limitations, such as the fact that it was a single-center study with a relatively small sample size, and no control group (without any blocks), in addition to the shorter duration of follow-up. Additional studies comparing different additives with different doses and concentrations of these blocks and examining the effect of varying block techniques on the post-operative outcome and the longer duration of post-operative follow-up will be valuable to verify these results. Also, in LAP group, there was no confirmation in the right position by US and cannot detect the spread of local anathestic drug in LTAP. Further studies need to compare different types, volumes and concentrations of local anesthetic with local infiltration and other blocks.

## Conclusion

In LAP bariatric surgery, the analgesic effect of LTAP is non-inferior to UTAP in terms of time to 1st rescue analgesia, total morphine consumption, and safety blocking through low post-operative complications and patient satisfaction. Thus, LATP and UTAP can be used whenever accessible.

## Data Availability

The datasets used and/or analyzed during the current study are available as MS Excel files (.xlsx) from the corresponding author upon reasonable request.

## Abbreviations

LAP: Laparoscopic
IV: Intravenous
TAP: Transversus Abdominis Plane
ERAS: Enhanced Recovery After Surgery
US: Ultrasound
TAM: Transversus Abdominis Muscle
LTAP: Laparoscopic Transversus Abdominis Plane
UTAP: Ultrasound Transversus Abdominis Plane
ASA: American Society of Anesthesiologists
BMI: Body mass index
VAS: Visual Analogue Scale
ECG: Electrocardiogram
NIBP: Noninvasive blood pressure
HR: Heart rate
MAP: Mean arterial pressure
PACU: Post-Anethesia Care Unit
PONV: Post-operative nausea and vomiting
SPSS: Statistical Package for Social Science
CI: Confidence interval
MABP: Mean Arterial Blood Pressure
